# The Global Use of Artificial Intelligence in the Undergraduate Medical Curriculum: A Systematic Review

**DOI:** 10.7759/cureus.39701

**Published:** 2023-05-30

**Authors:** Jonny R Varma, Sherwin Fernando, Brian Y Ting, Shahrukh Aamir, Rajesh Sivaprakasam

**Affiliations:** 1 Undergraduate Medical Education, Barts and The London School of Medicine and Dentistry, London, GBR; 2 Renal Transplant, Royal London Hospital, London, GBR

**Keywords:** systematic review, undergraduate medical education, medical students, education, artificial intelligence, medical education

## Abstract

Artificial intelligence (AI) is a rapidly advancing technology that has the potential to revolutionize medical education. AI can provide personalized learning experiences, assist with student assessment, and aid in the integration of pre-clinical and clinical curricula. Despite the potential benefits, there is a paucity of literature investigating the use of AI in undergraduate medical education. This study aims to evaluate the role of AI in undergraduate medical curricula worldwide and compare AI to current teaching and assessment methods.

This systematic review was conducted using the Preferred Reporting Items for Systematic Reviews and Meta-Analyses (PRISMA) reporting guidelines. Texts unavailable in English were excluded alongside those not focused on medical students alone or with little mention of AI. The key search terms were “undergraduate medical education,” “medical students,” “medical education,” and “artificial intelligence.” The methodological rigor of each study was assessed using the Medical Education Research Study Quality Instrument (MERSQI).

A total of 36 articles were screened from 700 initial articles, of which 11 were deemed eligible. These were categorized into the following three domains: teaching (n = 6), assessing (n = 3), and trend spotting (n = 2). AI was shown to be highly accurate in studies that directly tested its ability. The mean overall MERSQI score for all selected papers was 10.5 (standard deviation = 2.3; range = 6 to 15.5) falling below the expected score of 10.7 due to notable weaknesses in study design, sampling methods, and study outcomes.

AI performance was synergized with human involvement suggesting that AI would be best employed as a supplement to undergraduate medical curricula. Studies directly comparing AI to current teaching methods demonstrated favorable performance. While shown to have a promising role, there remains a limited number of studies in the field, and further research is needed to refine and establish clear foundations to assist in its development.

## Introduction and background

Artificial intelligence (AI) is the concept of machine-based computational functionality with an element of rationality in its processes, a term first coined in 1956 [1]. The term can be defined through various means, namely, between performance similar to that of humans, and performance that is deemed perfect or ideal (Table 1). Given the multiple definitions available for AI, the one that applies most in this context would be associating AI with thinking and acting rationally.

**Table 1:** The various definitions of artificial intelligence (AI).

Thinking humanly	Thinking rationally	Acting humanly	Acting rationally
“The exciting new effort to make computers think… machines with minds, in the full and literal sense” [1]. “[The automation of] activities that we associate with human thinking, activities such as decision-making, problem-solving, learning…” [2]	“The study of mental faculties through the use of computational models” [3]. “The study of the computations that make it possible to perceive, reason, and act” [4]	“The art of creating machines that perform functions that require intelligence when performed by people” [5]. “The study of how to make computers do things at which, at the moment, people are better” [6]	“Computational Intelligence is the study of the design of intelligent agents” [7]. “AI… is concerned with intelligent behaviour in artefacts” [8]

AI has already found a place in data analysis and informatics through the collection and analysis of data at volumes and speeds not humanly practical - these techniques are already being implemented in the educational setting [2,3]. Machine learning algorithms are used in schools for marketing, personalized recommendations, and managing course loads for students [4]. The development of Intelligent Tutoring Systems (ITS) from computer-based training and computer-aided instruction has become more prominent with the advancement of AI [5]. Thus, according to recent studies, ITS may have a minor favorable effect when compared to traditional classroom teaching [6]. Neural networks (NNs) are more contemporary designs of AI inspired by their biological counterparts. At their most basic, NNs are formed by input, hidden, and output layers with data freely passing through each layer by way of interconnected artificial neurons. NNs are adaptive systems and undergo a learning phase to determine a correlation between the input and output parameters [7]. Although such technology has been studied for use in clinical disciplines, its application in medical education is yet to be completely explored.

The term undergraduate within this study refers to medical students who have yet to achieve their primary medical qualification. The possibility of AI contributing to the development of medical education in general is now being explored [2,9]. However, there are no focused systematic reviews investigating the use of AI in the undergraduate medical curriculum. Previously, the lack of AI development in the medical setting was due to a variety of factors, including technical difficulties associated with its implementation, which would necessitate the collaboration of various specialists from various fields, such as data scientists, to optimize its performance in the medical setting [10]. Despite a surge in interest over the past decade, there is significant global inequality in the uptake and academic interest of these new technologies.

Methods of teaching students pre-clinical medicine (typically the first two years of the medical curriculum, encompassing basic sciences) have been described as inflexible, and educators face the challenge of integrating the pre-clinical and clinical curriculum [11]. AI has the potential to encourage this transition in the form of virtual, interactive patient cases and enhanced bedside teaching. A potential advantage of AI is its ability to provide one-on-one teaching with minimal consumption of human resources. While small group teaching is employed to mitigate this problem, AI may be a solution to allow for more tailored learning.

Student assessment in medical education has long been a contentious topic, as it serves as a crucial determinant of professional practice. The methods used to validate assessments in medical education vary significantly, encompassing diverse theoretical frameworks for validation. This heterogeneity highlights the need for standardized quality assurance in student assessment, considering its significant impact on overall student performance. To facilitate the increased involvement of AI in enhancing student assessment, a number of factors need rigorous consideration. Financial implications associated with implementing AI solutions and the handling of personally identifiable information are among the key concerns [2]. To harness the potential benefits of AI in this field, it is essential to navigate these considerations and establish robust frameworks for standardization, ensuring the ethical and secure use of AI in student assessment.

This study aims to evaluate the role of AI in undergraduate medical curricula worldwide and compare AI to the teaching and assessment methods currently employed by medical schools. This may provide a discriminatory platform for whether AI should be used in undergraduate medical curricula and its possible applications.

## Review

Methods

Search Design

This systematic review was conducted using the Preferred Reporting Items for Systematic Reviews and Meta-Analyses (PRISMA) reporting guidelines. Key Medical Subject Headings (MeSH) search terms used were “Undergraduate Medical Education” OR “Medical Student” AND “Artificial Intelligence.” The following databases were searched: PubMed, EBSCOhost Education Resources Information Center (ERIC) and Education Source, and Web of Science from inception to March 2021. A review protocol was established a priori. The target population was defined as undergraduate medical students. We ensured that all study participants had yet to complete their primary medical degrees, including students with and without previous degrees.

AI was defined as any system which was able to carry out a process otherwise performed by a human within the context of education. This included mathematical features of AI such as machine learning and computational elements of AI such as NNs and knowledge bases [1]. This broad definition allowed us to have a wide-angle view of the application of AI within all facets of the undergraduate medical curriculum.

Inclusion and Exclusion Criteria

Exclusion criteria were as follows: (1) full texts of articles that were unavailable in English, (2) articles with passing mentions of AI and that only suggested the use of AI in the future, and (3) Articles with education not explicitly focused on medical students. Inclusion criteria were articles with key or MeSH terms precisely resembling “undergraduate medical education,” “medical students,” "medical education,” and “artificial intelligence.”

Selection of Articles and Data Extraction

Titles and abstracts were reviewed by two co-authors (JTSF, JRV), and any duplicates were removed. Full-text screening of these articles was then performed independently by two reviewers (BYZT, SA) against the predefined eligibility criteria. Any discrepancies regarding the eligibility of an article were discussed, and a consensus was achieved by the third and fourth reviewers (JTSF, JRV). Two reviewers (BYZT, SA) independently extracted data, particularly the key parameters of AI applications from each respective study. Data regarding the lead author, year of publication, study design, outcome measures, number of participants, intervention, and comparator/control were extracted. A third and fourth reviewer (JTSF, JRV) independently repeated the process to verify the data. The study team also extracted data on relevant study characteristics to allow an assessment of study quality. Due to a paucity of literature, we were unable to conduct a formal meta-analysis and have, therefore, provided a narrative review of the findings.

Assessment of Study Quality

The quality of the study methodology was assessed by the Medical Education Research Study Quality Instrument (MERSQI), a validated scoring system for evaluating studies in medical education. Four co-authors (SA, JTSF, BYZT, JRV) individually reviewed each study’s MERSQI score to minimize bias. The MERSQI score ranges from 5 to 18, with higher scores predicting a higher quality study methodology. A score of 10.7 or above was considered to be a study of high quality [12,13].

Results

Study Selection

**Figure 1:**
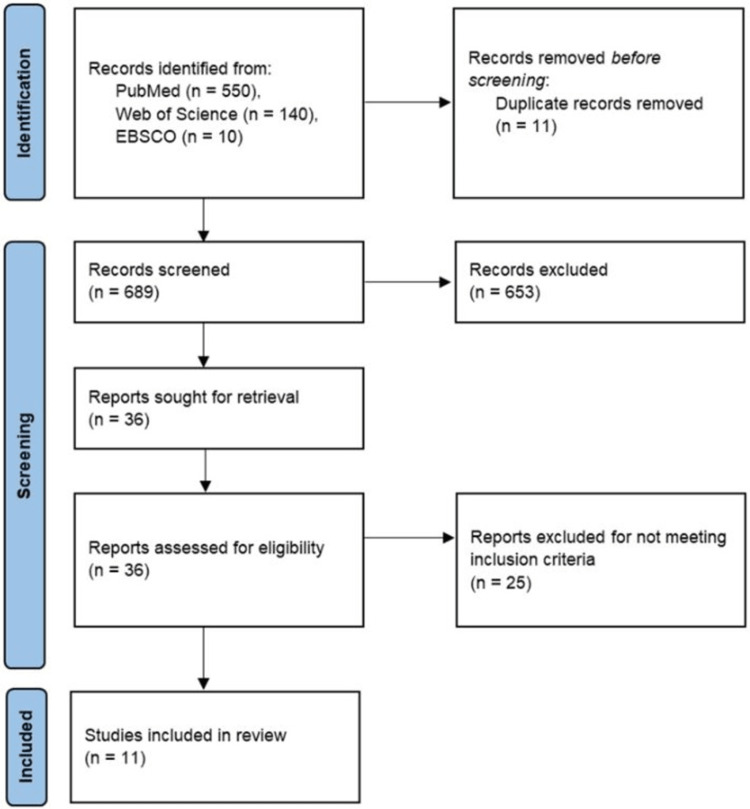
Preferred Reporting Items for Systematic Review and Meta-Analysis flowchart.

Our search yielded 700 English-language texts from the three aforementioned databases, of which 11 articles were duplicates. The remaining 689 texts underwent a keyword assessment for suitability. Many articles were excluded due to a lack of relevance to the target population (medical students) or the intervention (AI). The full texts of the remaining 36 articles were reviewed by the same co-authors. A total of 11 studies were included in the final results, utilizing AI within trend spotting [14,15], teaching [16-21], and assessing [22-24] (a PRISMA flowchart is provided in Figure 1).

Study Characteristics

Study groups: Table 2 provides a summary of the characteristics of the included studies. The articles were grouped into the following three domains: trend spotting, teaching, and assessing. This was based on their principal role in the context of medical education and defined to establish a basis for comparison. Trend spotting in the context of this review is defined as the use of AI to recognize patterns in the performance or characteristics of medical students as a method of providing feedback or to note trends within the entire cohort. Teaching is defined as the active usage and engagement of AI with medical students for didactic and instructional roles that would traditionally be undertaken by lecturers or other qualified personnel. Assessing is defined as the use of AI to oversee or evaluate medical students’ examination performance against a predetermined set of values or standards.

**Table 2:** Summary of the study outcome data. AAMC: American Association of Medical Colleges; AI: artificial intelligence; CIRCSIM-tutor: a language-based intelligent tutoring system; MERSQI: Medical Education Research Study Quality Instrument; MLA: machine learning algorithm; SOM: self-organized map; TTE: transthoracic echocardiography; VR: virtual reality; VP: virtual patient

Domain	Author	Date	Discipline(s)	Country	Primary outcomes	Results	Mean MERSQI score (/18)
Trend spotting	Stevens, et al. [14]	1994	Pre-clinical Medical Education, Immunology	United States	Detecting the diagnostic accuracy of student and clinician performances using artificial neural networks	Correct classification for students occurred more than 85% of the time. Recognition of clinician performances was poor, as low as 13%. The results between these two groups were significantly different (p < 0.0005)	12
	Delzell Jr et al. [15]	2009	Pre-clinical Medical Education, General	United States	Use of an artificial neural network to determine if medical students’ information-gathering patterns formed clusters of similar strategies, and if so to calculate the percentage of incorrect diagnoses in each cluster	SOM provided seven clusters of information-gathering patterns, with the percentage of incorrect diagnoses differing significantly among these clusters (range = 0-42%, p = 0.034)	11
Teaching	Persad, et al. [16]	2016	Medical Education, General	Canada	Survey of students and clinicians who used an AI VP simulator	Program was described as “a significant improvement” over software that the cohort had previously used. Free-text input provided a personalized learning experience while also challenging the subjects more than other software had done in the past	8
	Khumrin et al. [17]	2017	Medical Education, General	Australia	Using student log cases and electronic health cases to verify the ability of neural networks to accurately diagnose a case of abdominal pain. The model was also used to provide personalized feedback to medical students	Logitboost and Naïve Bayes were the most accurate neural network classifiers reaching the correct differential diagnoses 94.7% and 85.1% of the time, respectively	11
	Khumrin et al. [18]	2018	Medical Education, General	Australia	Use of the DrKnow system, a web-based AI learning application that presents students with personalized feedback and evaluation about their overall diagnostic performance, similar to the role of an expert clinician. Two clinically similar patient scenarios were selected (appendicitis and ectopic pregnancy) and the performance of DrKnow was evaluated	DrKnow was able to identify the proper diagnosis regarding the clinical findings in both scenarios, with appropriate identification of the differences between the two cases. 90% (n = 9) of the appendicitis and 70% (n = 7) ectopic pregnancy clinical findings were correctly identified by DrKnow	12
	Gorby. et al. [19]	2001	Pre-clinical Medical Education, Microbiology	United States	Comparison of student feedback of the same AI-enhanced lecture and non-AI-enhanced lecture	18% increase (3.85 to 4.85) in positive feedback from Creighton students and 21% increase (3.13 to 3.79) in positive feedback from Nebraska students	7
	Micheal et al. [20]	2003	Pre-clinical Medical Education, Cardiac Physiology	United States	Comparison of a pre-test/post-test after integrating CIRCSIM-Tutor into a cardiac physiology lesson focused on the baroreceptor reflex. This AI program is a computer tutor designed to carry out a natural language dialogue with a medical student	Students were able to correctly describe more of the relationships between system variables, with an 18% improvement (13.64 to 16.16, p < 0.001) between pre-test and post-test scores. There was a 32% improvement (2.24 to 2.96, p < 0.001) in multiple-choice question test scores after the use of CIRCSIM-Tutor. The total number of misconceptions decreased by 55% (4.07 to 1.83, p < 0.001) after using CIRCSIM-Tutor. Students in the setting with an instructor showed the greatest improvement. On 9 out of 10 questions in the survey, students agreed that CIRCSIM-Tutor was helpful	13
	Bric et al. [21]	2014	Medical Education, Surgical Skills	United States	Comparison of the completion of two Fundamentals of Laparoscopic Surgery tasks before and after teaching through a VR stimulator	Following completion of VR training, scores improved by 26% (175.5 to 220.9, p < 0.001) in peg transfer and 418% (20.2 to 104.7, p < 0.001) in intracorporeal knot tying. The percentage of successful knots improved by 131% (27 to 63, p < 0.01)	13
Assessing	Chen et al. [22]	2014	Medical Education, Geriatric Medicine	United States	Use of three MLAs to identify student experiences in six AAMC geriatric competencies (medication management, cognitive and behavioral disorders, falls, balance, gait disorders, self-care capacity, palliative care and care for elders) from their clinical notes	The mean Fmeasure score (score representing precision, with 1.00 representing 100% precision) of the three MLAs across the six domains was 0.80 (standard deviation = 0.12)	10
	Spickard et al. [23]	2014	Medical Education, General	United States	To validate an AI scoring system that rates medical students’ clinical notes for relevance to priority topics of the medical school curriculum to establish progress toward institutional competency goals	Upon assessing 16 core clinical topics (i.e., abdominal pain, chest pain), a positive predictive value of at least 75% or higher was achieved in each of the domains, with the highest value being 83.3% (score = 0.8, 95% confidence interval = 0.73–0.88).	10
	Langet et al. [24]	2020	Medical Education, Cardiology	France	Comparison of performance in standard TTE with AI-assisted TTE relative to a reference TTE done by an expert	70% of medical students performed better with AI assistance (pre-score = 60.8, post-intervention score = 81.7, p < 0.001). TTEs with AI assistance were also 27.5% more suitable for clinical use	9

MERSQI scoring: The overall MERSQI scores of all selected studies ranged from 6 to 15.5. The mean overall MERSQI score for all selected papers was 10.5 (standard deviation (SD) = 2.3). Research methodologies for “trend spotting” papers scored 11.6 (SD = 1.1), “teaching” scored 10.6 (SD = 2.6), and “assessment” scored 9.6 (SD = 1.8). AI research methodologies tended to use objectively measured data, valid evaluation instruments, and equitable statistical methods. However, particular points of weakness in the literature (defined as a domain scoring 1.5 points or less) were the study design (median score of 1 out of 3), sampling methods (median score of 1.5 out of 3), and outcome measurements (median score of 1 out of 3). The mean overall MERSQI scores assigned to papers under the “trend spotting” domain were higher than those under the “assessment” domain (p<0.05). No significant difference was found between the “teaching” and “assessment” domains.

Trend spotting: A total of two trend-spotting studies were found, one directed at clinical medical students and the other at pre-clinical medical students. One demonstrated the ability to distinguish medical students’ performance markedly better than experienced clinicians, suggesting a characteristic difference between strategies employed by the different parties [14]. The second study used AI to analyze information-gathering patterns in medical students and identified clusters that performed worse with a different weighting of specific steps, such as physical examination [15].

Teaching: A total of six studies were identified within the teaching category. Studies in the teaching and assessing domains were experimental trials, predominantly considering the use of AI in NNs for virtual patient cases. One particular stimulator allowed subjects to input their text into the software, giving them a more personalized experience [16]. NNs were shown to be effective with accuracies of up to 94.7% [17,18]. Commercial robots such as the Verbot system [19], CIRCSIM Tutor [20], and virtual reality (VR) [21] all yielded statistically significant positive results regarding student satisfaction and effectiveness (measured using a pre-test and post-test system).

Assessing: All three studies in the assessment domain were focused on the clinical aspect of the medical curriculum. Two studies [22,23] explored the use of AI in notes made by students examining patients while on placement by evaluating them against selected competency domains. Clinicians also scored the students in the same fields, and these scores were compared to determine the accuracy of the AI. The final study considered AI applications in transthoracic echocardiography (TTE) [24], showing a 70% improvement in performance with AI assistance (20.1 score increase, p < 0.001).

Discussion

The results indicated that AI was used in a variety of specialties within undergraduate medical education, with a skewed emphasis on the clinical aspects of the curriculum [17,18,21-24] rather than the pre-clinical [14,15,19,20]. Trend spotting and teaching programs were used equally for pre-clinical and clinical audiences. However, AI in assessment was primarily aimed at a clinical audience. This is likely due to the homogenous structure of the pre-clinical curriculum with its focus on learning concepts compared to the clinical curriculum which has a greater focus on the application of concepts, decision-making, and practical skills.

AI as a Trend-Spotting Tool

AI has been shown to have a role in tailoring specific curriculum components to students’ needs. By identifying trends and highlighting knowledge deficits, students may receive personalized feedback on both their answers and their thinking process. With AI’s ability to spot trends in student performance, it can ameliorate the difficulty of a human instructor to respond to every student in a practical manner.

AI has also demonstrated the ability to categorize medical students’ performance more efficiently than that of clinicians. NNs were trained on previous students’ successful problem performances, which suggests that clinicians approach problems differently due to well-developed skills and experience [14]. Understanding this difference would play a central role in equipping the clinical medical students of the future. Fluency in clinical history, examination, and decision-making process demonstrates the most variability between different experience levels. Such patterns of thinking may be identified and clustered into similar information-gathering trends among students [15]. The best-performing cluster selected 2.5 times more items than the worst-performing cluster, with 4.4 times more items relating to the patient’s past medical history and 2.6 times more items relating to the physical examination. Therefore, the model suggests that an investigation of more items tends to result in fewer percentages of incorrect diagnoses. Naturally, this must be balanced with real-life pragmatism and acumen.

AI in Teaching

AI implementation was associated with higher levels of positive student feedback. There was a 32% improvement (2.24 to 2.96, p < 0.001) in multiple-choice question test scores after the use of CIRCSIM compared to didactic teaching alone due to teaching quality and student engagement. The CIRCSIM tutor showed that students performed best when using the program alongside an instructor [20]. This implies that, while AI implementation may be beneficial in pre-clinical medical education, an expert’s position will not be rendered obsolete.

The authors believe that AI has a future role in simulated bedside teaching, skills work, and anatomy dissection. AI can be combined with VR devices to create a realistic, yet safe environment to practice both acute emergency scenarios and simulated operations [25]. Mixed reality devices with integrated AI programs have already demonstrated the ability to recognize anatomical landmarks in clinical practice to provide operators with extensive clinical information [9]. Automated anatomical landmark recognition has the potential for both anatomical education and dissection for future undergraduate cohorts.

AI in Assessment

As students progress from the lecture hall to the clinical stage of their studies, their assessments become more centered on their clinical performance. Machine learning algorithms can identify student performances from the completeness of their clinical notes [22], and AI may also be used to evaluate the performance of imaging interpretation including TTEs, which resulted in a 70% improvement (pre-score = 60.8, post-intervention score = 81.7, p < 0.001) in performance compared to human assessment alone [24]. This study corroborates previous studies [21,22] that show AI assistance is beneficial for medical students in the clinical stage of the medical curriculum.

The authors recognize that AI has been employed in the assessment of clinical activities by students. There is a growing body of evidence demonstrating the significant difficulties faced in medical examination by ethnic minority undergraduates and postgraduates [26]. The authors, therefore, implore educational bodies to look to more innovative and inherently objective methodologies such as AI as a potential solution to this problem.

Unanswered Challenges Facing Implementation

Implementing AI in the medical curriculum is not without its drawbacks, with many ethical concerns remaining unsolved. Given the inherently unknown AI mechanisms, any automated changes to the educational system may pass without appropriate scrutiny, possibly leading to inappropriate recommendations for students. There are also legal ramifications involving the consent and availability of personally identifiable information [2].

The implementation of new technology comes with the inevitable financial burden, and AI-based medical education tools are no different. Various challenges face medical education in developing countries, including constraints on government budgets, meaning universities are forced to take on more fee-paying students [27]. There have already been various successful attempts to use AI as a low-cost, time-efficient, high-impact initiative to strengthen the training of healthcare professionals in developing countries such as Somalia [28] and Malaysia [29].

Though AI programs are available commercially, they often require expensive institutional licenses to be utilized in the classroom. The authors acknowledge that the implementation of AI within the undergraduate curriculum of developing countries will no doubt be hindered by the financial burden. Achieving synergy between a human tutor and an AI tool requires additional training to facilitate the appropriate usage of technology and the basic know-how for common technical errors. This additional level of training serves as a further hurdle to educators in the developing world.

Study limitations

Although AI is an enthralling field, the methodological rigor of AI research must uphold sufficient standards before AI can be considered for integration into the undergraduate medical curriculum. The eligible studies’ overall mean MERSQI score was 10.5, falling below the expected standard of 10.7 for studies of high quality in medical education research. Hence, the findings imply that although the present AI research is ambitious, the studies lack vital elements. The recurring weaknesses identified in the current literature were the study design, sampling methods, and study outcomes.

MERSQI defines the gold standard of educational study design as being a randomized control trial. However, all eligible studies used cross-sectional or observational designs [14-24]. AI programs were often applied as one-off sessions without significant long-term usage or follow-up and were not reliably tested as an integrated element of their respective curricula. Information bias is prevalent in educational studies due to the difference in experience level and agenda between students and supervisors. Additionally, most studies did not have well-defined eligibility criteria; for example, many student cohorts participated as volunteers, thus introducing selection bias. Only two studies [14,19] expanded their program participation over multiple institutions, which improved study reliability by reducing selection bias. Study outcomes tended to focus more on knowledge, skills, and user satisfaction, and less so on patient-centered outcomes. The MERSQI scoring system has demonstrated certain limitations in this study. There is no standardized interpretation of the scale of MERSQI scores; previous studies have relied on arbitrary interpretations of the scale [30].

## Conclusions

AI has a promising role in the progression of the undergraduate medical curriculum to nurture tomorrow’s doctors in the technological era of today. However, due to the limited number of studies available for analysis, the scope for evaluating the impact of AI remains restricted. Furthermore, no study has achieved 100% accuracy, highlighting the necessity for continued development of this technology. While general applications in medical education have been identified with performances equalling to or exceeding humans, further research is needed to refine and establish clear foundations to assist in the development. While AI can substantially change the medical curriculum in its current form, the authors stress that AI should be used as a supplement within the undergraduate medical curriculum; ultimately, there is no better preparation for a student than facing real patients with real medical problems.
